# Discordance between microvascular permeability and leukocyte dynamics in septic inducible nitric oxide synthase deficient mice

**DOI:** 10.1186/cc6190

**Published:** 2007-12-07

**Authors:** Steven M Hollenberg, Massimiliano Guglielmi, Joseph E Parrillo

**Affiliations:** 1 Cooper University Hospital, Cooper Plaza, Camden, New Jersey 08103, USA

## Abstract

**Introduction:**

Microvascular dysfunction causing intravascular leakage of fluid and protein contributes to hypotension and shock in sepsis. We tested the hypothesis that abrogation of inducible nitric oxide synthase (iNOS) activation would decrease leukocyte rolling, leukocyte adhesion, and microvascular leakage in sepsis. We compared wild-type mice made septic by cecal ligation and puncture with mice deficient in iNOS.

**Methods:**

Leukocyte dynamics and microvascular permeability were assessed simultaneously by fluorescence intravital microscopy in the cremaster muscle 15 to 20 hours after induction of sepsis by cecal ligation and puncture in C57Bl/6 mice. Rolling and adhesion of leukocytes labeled with rhodamine and leakage of fluorescein isothiocyanate-conjugated albumin was measured in single nonbranching venules (25 to 40 μm) and compared among septic wild-type, septic iNOS-deficient transgenic, and sham-operated control mice.

**Results:**

Leukocyte rolling and adhesion were increased in septic animals (61.6 ± 14.4 cells/minute and 4.1 ± 0.6 cells/100 μm per minute, respectively) as compared with control animals (8.5 ± 2.3 cells/minute and 1.1 ± 0.2 cells/100 μm per minute, respectively; *P *< 0.001 for both). Rolling increased in iNOS-deficient septic mice (to 105.5 ± 30.0 cells/minute, *P *= 0.048, versus wild-type septic); adhesion was unchanged (5.1 ± 0.5 cells/100 μm per minute, *P *= 0.30). Sepsis produced an increase in leakage ratio in wild-type septic mice compared with controls (0.36 ± 0.05 versus 0.08 ± 0.01, *P *< 0.001). Leakage was attenuated in iNOS-deficient septic mice (0.12 ± 0.02, *P *< 0.001, versus wild-type septic mice).

**Conclusion:**

Leukocyte adhesion and vascular leakage were discordant in this setting. The finding that septic iNOS-deficient mice exhibited less microvascular leakage than wild-type septic mice despite equivalent increases in leukocyte adhesion suggests an important role for nitric oxide in modulating vascular permeability during sepsis.

## Introduction

The most important pathophysiological abnormalities in sepsis and other severe inflammatory conditions occur at the microvascular level. These abnormalities include persistent vasodilation refractory to vasopressors, activation of leukocytes resulting in oxidative stress, inflammation, and the potential for capillary plugging, increased microvascular leakage, platelet activation and microthrombus formation, and microvascular shunting.

The postcapillary venules are the primary site of inflammatory events, which include neutrophil adhesion and emigration as well as protein and water leakage. Endothelial-directed recruitment and activation of neutrophils at the site of infection to eradicate pathogens is a central feature of the innate immune response to infection. The upregulation of adhesion molecules by proinflammatory mediators becomes widespread in severe sepsis, occurring not only at the site of infection but also throughout the vasculature. As such, neutrophils can adhere to and damage endothelium in noninfected tissues, contributing to the multiorgan failure characteristic of severe sepsis [1,2].

Many of the effects of inflammatory cytokines elaborated during sepsis are mediated through nitric oxide (NO), which is an important regulator of vascular tone, leukocyte adhesion to microvascular endothelium, and capillary leakage. Activation of the cytokine-inducible nitric oxide synthase isoform (iNOS), with consequent over-production of NO, has been well documented in both animal models of sepsis and in septic patients, and leads to vasodilation and pressor refractoriness [3-6]. Recent investigations have suggested that iNOS activity may be compartmentalized at the site of infection and parallels expressions of inflammatory cytokines [7]. Endothelium-derived NO produced by the constitutive NOS isoform, however, is an important endogenous inhibitor of leukocyte adhesion to the microvascular endothelium [8].

We hypothesized that abrogation of iNOS activation would decrease leukocyte rolling, leukocyte adhesion, and microvascular leakage in sepsis. To test this hypothesis, we compared wild-type mice made septic by cecal ligation and puncture (CLP) with knockout mice deficient in iNOS.

## Materials and methods

The study was performed in accordance with US National Institutes of Health (NIH) guidelines for the use of experimental animals, and the protocol was approved by the institutional Animal Care and Use Committee. Animals were made septic by cecal ligation and puncture, and microvascular responses were assessed using *in vivo *videomicroscopy.

### Cecal ligation and puncture

Sepsis was induced surgically by CLP as previously described [9,10]. Wild-type C57/BL6 and iNOS-deficient transgenic C57/BL6 mice [11] were anesthetized for laparotomy. The cecum was ligated and punctured with an 18-gauge needle. For sham operations, laparotomy was performed but ligation and puncture omitted. Animals were given normal saline 100 ml/kg subcutaneously after the procedure.

### Videomicroscopy

Mice were prepared for videomicroscopic observations 12 to 15 hours after CLP. The mice were anesthetized with inhaled isoflurane and the carotid artery cannulated for measurement of blood pressure and intra-arterial infusion. The mice were pretreated with cromolyn sodium 5 mg/kg intra-arterially to prevent mast cell degranulation and histamine release [12].

The cremaster muscle was dissected and exteriorized onto an optically clear viewing platform with blood and nerve supplies preserved and suffused with physiologic Krebs solution [10,13]. The preparation was placed on a custom-designed platform on the stage of an upright microscope and the transilluminated microcirculation was viewed through a 40× objective. The image was projected by videocamera onto a monitor and recorded on a video cassette recorder. Single unbranched postcapillary venules (20 to 40 μm in diameter, 250 μm long) were selected for study. Venular diameter was measured off-line using a frame grabber and NIH-Image analysis program (NIMH, Bethesda, MD, USA). Mean red blood cell velocity was measured using an optical Doppler velocimeter (Microcirculation Research Institute, Texas A&M University, College Station, TX, USA), which uses a pair of photodiodes to generate a voltage from an image of moving red cells that is a linear representation of red cell velocity [14]. Wall shear rate was calculated based on the Newtonian definition as (mean red blood cell velocity/diameter) × 8 (seconds^-1^) [15].

### Experimental protocol

After the preparation was in place, 60 minutes were allowed for it to reach a steady state. Single unbranched postcapillary venules (20 to 30 μm in diameter, 250 μm long) were selected for study. Leukocytes were labeled with rhodamine 6G (5 mg/kg) given intra-arterially to facilitate visualization, and imaged with a rhodamine cube in a Nikon E600 fluorescence microscope. The number of rolling and adherent leukocytes was determined by offline playback of videotaped images. Leukocytes were considered to be rolling if they were moving more slowly than red blood cells. The rolling rate was expressed as the number of cells moving past a designated point per minute (leukocyte flux) [8,15]. A leukocyte was defined as adherent to venular endothelium if it remained stationary for longer than 30 seconds. Adherent cells were expressed as the number per 100 mm length of the venule per minute [12,16,17].

To quantify albumin leakage across cremasteric postcapillary venules, 50 mg/kg fluorescein isothiocyanate-labeled albumin (Sigma Chemical Co, St Louis, MO, USA) was administered intra-arterially and fluorescence intensity detected using a SIT camera (Hamamatsu, Hamamatsu City, Japan). The fluorescence intensity of fluorescein isothiocyanate-albumin within three segments of the venule under study (V_i_) and in three contiguous areas of perivenular interstitium (V_o_) equally spaced from the midline of the vessel (at 40 μm and 60 μm on each side) was measured at 10 minutes, averaged, and leakage indexed as V_o_/V_i _[18].

Four groups of animals were studied: sham-operated control mice (*n *= 8), sham-operated iNOS-deficient mice (*n *= 8), wild-type mice made septic by CLP (*n *= 8), and iNOS-deficient mice made septic by CLP (*n *= 10).

### Selective iNOS inhibition

To further evaluate the role of iNOS in inflammation, leukocyte adhesion, and microvascular leakage in sepsis, mice were treated with the selective iNOS inhibitor 1400W. Mice were made septic by CLP and resuscitated with fluids and antibiotics as described above. 1400W (10 mg/kg) was given intramuscularly at the time of CLP, 6 hours later, and 12 hours later, and videomicroscopy was then performed.

### Materials

Cromolyn and 1400W were obtained from Sigma Chemical Co. Appropriate dilutions were made with modified Krebs solution.

Transgenic mice deficient in iNOS were obtained from The Jackson Laboratory (Bar Harbor, ME, USA). These mice were shown to lack detectable iNOS mRNA and iNOS protein, and not to produce NO (as detected by serum nitrate/nitrite levels) after endotoxin stimulation [11].

### Data analysis

Data are expressed as mean ± standard deviation, with *n *indicating the number of animals. In each experimental animal, only one vessel was tested. Statistical testing was done using one-way analysis of variance for group comparisons and Tukey HSD () honestly significantly different) for post-analysis of variance comparisons or unpaired Student's *t*-tests for single comparisons. P < 0.05 was deemed statstically significant.

## Results

The bacteriology and mortality associated with this septic model, which is designed to replicate the main clinical modalities used in septic patients, have been reported previously; mice become bacteremic with Gram-negative rods and anaerobic organisms [19]. Mortality in unresuscitated Balb/C mice was 100%, and decreased to 80% with fluid resuscitation only, to 72% with antibiotics only, and to 54% with both fluids and antibiotics (*P *< 0.01 by Kaplan-Meier analysis) [19].

Mean arterial pressure was lower in wild-type mice after CLP (76 ± 10 mmHg) than in sham-ligated control wild-type animals (90 ± 6 mmHg; *P *< 0.05). Mean arterial pressure in iNOS-deficient control animals was 94 ± 5 mmHg, a value not different from that of wild-type controls. Mean arterial pressure in septic iNOS-deficient mice was 86 ± 10 mmHg, which did not differ significantly from that of either wild-type or iNOS-deficient controls.

Circulating white blood cell (WBC) counts were decreased in septic wild-type mice compared with sham-operated controls (0.9 ± 0.1 × 10^6 ^cells/ml versus 1.7 ± 0.2 × 10^6 ^cells/ml; *P *< 0.05). In iNOS-deficient control mice, WBC counts were slightly but not significantly higher than in wild-type controls (2.0 ± 0.4 × 10^6 ^cells/ml), and were also lower in septic mice (to 1.1 ± 0.3 × 10^6 ^cells/ml, *P *< 0.05, versus control), but WBC counts in iNOS-deficient septic mice did not differ significantly compared with wild-type septic mice. Venular shear rates were slightly higher in septic wild-type (622 ± 63 seconds^-1^) and septic iNOS-deficient mice (661 ± 87 seconds^-1^) than in wild-type (610 ± 74 seconds^-1^) or iNOS-deficient control mice (618 ± 87 seconds^-1^), but these differences were not significant by analysis of variance (*P *= 0.33).

**Figure 1:**
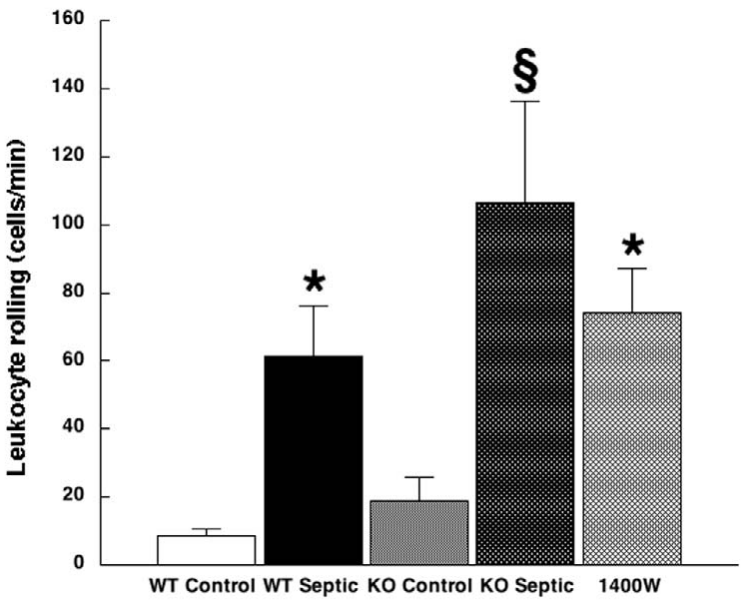
Leukocyte rolling. Shown is leukocyte rolling in wild-type control mice (white bar; 8.5 ± 2.3 cells/minute; *n *= 8), wild-type septic mice (black bar; 61.6 ± 14.4 cells/minute; *n *= 8), inducible nitric oxide synthase (iNOS)-deficient control mice (light stippled bar; 18.8 ± 6.9 cells/minute; *n *= 8), iNOS-deficient mice (dark stippled bar; 105.5 ± 30.0; *n *= 10), and mice treated with the selective iNOS inhibitor 1400W (cross-hatched bar; 74.0 ± 13.3 cells/minute; *n *= 5). *P < 0.001 versus wild-type control. ^§^*P *< 0.001 versus wild-type septic. KO, iNOS-deficient knockout; WT, wild-type.

Induction of sepsis increased microvascular leukocyte rolling in wild-type mice, from 8.5 ± 2.3 cells/minute in sham-operated controls to 61.6 ± 14.4 cells/minute in mice made septic by CLP (*P *< 0.001). Rolling was increased in iNOS-deficient control mice compared with wild-type controls (to 18.8 ± 6.9 rolling cells/minute; *P *= 0.048) and further increased in iNOS-deficient septic mice (105.5 ± 30.0 cells/minute, *P *< 0.001, versus both iNOS-deficient controls and wild-type septic mice). In mice treated with the iNOS inhibitor 1400W, rolling was 74.0 ± 13.3 cells/minute, a value significantly higher than controls that in (*P *< 0.001) but not significantly different from either wild-type or iNOS-deficient septic mice. See Figure 1.

**Figure 2:**
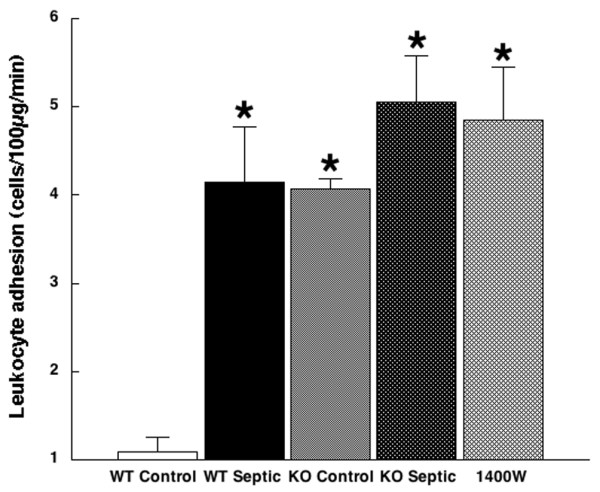
Leukocyte adhesion. Shown is leukocyte adhesion in wild-type control mice (white bar; 1.1 ± 0.2 cells/100 μm per minute; *n *= 8), wild-type septic mice (black bar; 4.1 ± 0.6 cells/100 μm per minute; *n *= 8); inducible nitric oxide synthase (iNOS)-deficient control mice (light stippled bar; 4.1 ± 0.1 cells/100 μm per minute; *n *= 8), iNOS-deficient mice (dark stippled bar; 5.1 ± 0.5 cells/100 μm per minute; *n *= 10), and mice treated with the selective iNOS inhibitor 1400W (cross-hatched bar; 4.9 ± 0.6 cells/100 μm/minute; *n *= 5). *P < 0.001 versus wild-type control. KO, iNOS-deficient knockout; WT, wild-type.

Sepsis also increased leukocyte adhesion in wild-type mice, from 1.1 ± 0.2 cells/100 μm per minute in sham-operated controls to 4.1 ± 0.6 cells/100 μm per minute in mice made septic by CLP (*P *< 0.001). Adhesion was increased in iNOS-deficient control mice compared with wild-type controls (to 4.1 ± 0.1 cells/100 μm per minute; *P *= 0.001) and also in iNOS-deficient septic mice (5.1 ± 0.5 cells/100 μm per minute, *P *< 0.001, versus wild-type septic mice, *P *= 0.30, versus iNOS-deficient controls) and after iNOS inhibition with 1400W (4.9 ± 0.6 cells/100 μm per minute, *P *< 0.001, versus wild-type septic mice). See Figure 2.

**Figure 3:**
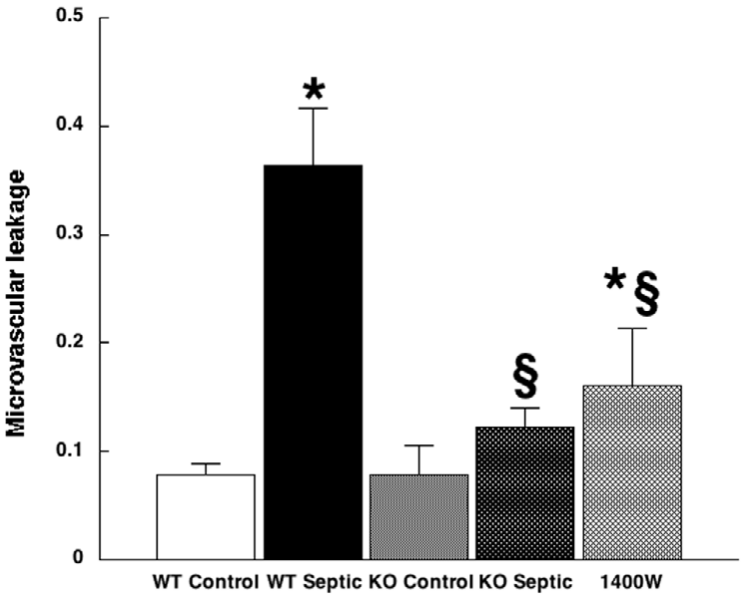
Microvascular leakage. Shown is microvascular leakage in wild-type control mice (white bar; 0.08 ± 0.01; *n *= 8), wild-type septic mice (black bar; 0.36 ± 0.52; *n *= 8), inducible nitric oxide synthase (iNOS)-deficient control mice (light stippled bar; 0.08 ± 0.03; *n *= 8), iNOS-deficient mice (dark stippled bar; 0.12 ± 0.02; *n *= 10), and mice treated with the selective iNOS inhibitor 1400W (cross-hatched bar; 0.16 ± 0.05; *n *= 5). *P < 0.001 versus wild-type control. ^§^*P *< 0.001 versus wild-type septic. KO, iNOS-deficient knockout; WT, wild-type.

Microvascular leakage, as assessed by leakage index, was similarly increased with sepsis in wild-type mice, from 0.08 ± 0.01 to 0.36 ± 0.52. Despite the increased leukocyte rolling and adhesion, microvascular leakage was not increased in iNOS-deficient controls (0.08 ± 0.03, P = 0.99, versus wild-type). When iNOS-deficient mice were made septic by CLP, the increase in microvascular leakage was substantially attenuated (0.12 ± 0.02, P < 0.001, versus wild-type septic mice, P = 0.08, versus iNOS-deficient controls). In mice treated with the iNOS inhibitor 1400W, leakage was also lower than in wild-type septic mice (0.16 ± 0.05; P = 0.02), but it was significantly higher than in sham-operated controls (P < 0.01). See Figure 3.

## Discussion

The main finding of the present study was a disjunction between leukocyte adhesion and vascular leakage; leukocyte rolling and adhesion was increased in septic mice both with and without iNOS induction, but microvascular leakage in septic mice was not increased in the absence of iNOS. This finding does not result from differences in circulating WBC counts, and is not explained by differences in macrocirculatory hemodynamic parameters, because vascular shear stress was unchanged, and increased blood pressure would not be expected increase the number of adherent leukocytes or decrease leakage. This suggests an important role for iNOS in modulating vascular permeability during sepsis independent of effects on leukocytes. These results have both mechanistic and therapeutic implications.

The vascular endothelium is one of the earliest targets of injury in inflammatory states, ultimately contributing to organ dysfunction and failure. Endothelial-directed recruitment and activation of neutrophils at the site of infection to eradicate pathogens is an important mechanism of the inflammatory response. Upregulation of complementary adhesion molecules and ligands on leukocytes and endothelial cells induced by bacterial products and proinflammatory mediators initiates a multistep process that includes initial contact between leukocyte and endothelium, followed by a weak transient adhesive interaction, manifested as leukocyte rolling, followed by firm leukocyte adhesion to the vessel wall [20]. Firm adhesion then allows leukocytes to transmigrate across the vessel wall to target sites [21]. Leukocytes, upon adherence to endothelial cells, become activated and generate reactive oxygen and nitrogen species, with the potential for endothelial damage [22]. This activation can propagate tissue injury, and its extent is predictive of outcome [23]. Both leukocyte activation and endothelial injury can increase microvascular permeability.

Endothelial barrier dysfunction in sepsis contributes to decreased preload in the initial phases, and to peripheral edema in later stages. Endothelial activation by inflammatory mediators leads to structural changes that increase perivascular permeability and to upregulation of adhesion molecule expression on the endothelial cell surface. Endothelial cells form the structural barrier to capillary leakage, while proteins such as protein kinase C and second messengers including cGMP provide functional aspects of this barrier. Both endothelial cell contraction, which involves actin-myosin interaction and changes in intracellular calcium, and passive cellular retraction, which probably involves protein kinase C phosphorylation and actin linking at intercellular tight junctions [24], can alter endothelial shape and can result in increased leakage. Such changes could be produced by inflammatory mediators such as tumor necrosis factor and interleukin-1 [25], or by leukocyte adhesion, with effects on the underlying cytoskeletal structure or activation and initiation of an oxidative burst. Leukocyte adhesion, however, is not strictly necessary for increased protein leakage during endotoxemia. In a study of rats given endotoxin by continuous infusion, fucoidin, a selectin-binding carbohydrate, blocked leukocyte adhesion but it did not significantly decrease leakage of albumin [26]. It seems probable that both leukocyte adhesion and circulating mediators play a role in mediating endothelial barrier dysfunction in sepsis, possibly with a different time course.

Constitutively produced NO normally regulates leukocyte recruitment, and its inhibition increases leukocyte rolling and adhesion [8]. Responses to the very high levels of NO that can be produced by iNOS are more complex and can be variable. Leukocyte rolling is generally increased in response to endotoxin challenge in mice deficient in iNOS [27], although the degree of adhesion differs in different models, with increased adhesion in iNOS knockout mice compared with wild-type mice with lower doses of endotoxin [27], equivalent adhesion with high-dose endotoxin [28], and decreased adhesion in CLP, at least in some organs [29]. The severity of the inflammatory insult thus appears to be an important determinant of leukocyte responses. Experiments with chimeric mice with either iNOS in leukocytes only (wild-type bone marrow transplanted into iNOS-deficient mice) or in parenchyma only (iNOS^-/- ^bone marrow transplanted into wild-type mice) challenged with endotoxin have revealed that in tissues other than the lung, parenchymal cells are the principal source of iNOS during endotoxemia, and parenchymal NOS is the dominant source of systemic iNOS activity. In the lung, however, endotoxin-induced iNOS is derived largely from infiltrating leukocytes [30]. As demonstrated by these studies, regional vascular responses in sepsis and inflammation can be heterogeneous. In this context, our hypothesis that iNOS-deficient septic mice would have decreased leukocyte adhesion compared with wild-type septic mice was not confirmed, but our finding of increased leukocyte rolling and equivalent leukocyte adhesion in iNOS-deficient knockout mice was in keeping with previous investigations. We also found increased leukocyte adhesion in iNOS-deficient control mice. The reason for this observation is uncertain, but it may suggest that, in the absence of iNOS, alternative mechanisms regulate leukocyte trafficking in response to the stress of cremaster dissection.

NO also modulates vascular permeability. Low levels of NO, such as would be expected from activation of the constitutive NOS isoform, in general decrease vascular permeability [31]. In fact, early nonselective inhibition of NOS after endotoxin challenge increased vascular permeability in a rat model [32]. On the other hand, when NOS inhibition was delayed until 3 hours after endotoxin in this model, such inhibition ameliorated the abnormal vascular leakage [32]. The idea that activation of the constitutive NO synthase isoform is protective but that higher levels can be damaging is a recurrent theme with particular resonance in sepsis pathogenesis; similar effects have been postulated for vascular tone [17] as well as myocardial contractility [33].

The findings of the present study suggest that iNOS is an important initiator of increased vascular permeability in sepsis. Selective iNOS inhibition with 1400W and induction of sepsis in iNOS-deficient mice both showed reduced vascular permeability without decreasing leukocyte adhesion. 1400W produced less attenuation of vascular leakage, probably because iNOS production was not entirely abrogated, although nonspecific effects of this inhibitor cannot be excluded. Nonetheless, the consistency of the findings with two different methods of attenuating iNOS production bolsters the evidence for the relevance of iNOS induction to vascular leakage. Demonstration of the importance of iNOS in a clinically relevant infectious model is important because much of the previous work in rodents has been done in the inflammatory endotoxin model. Unlike humans, rodents are resistant to endotoxin, and use of the high doses of endotoxin necessary to produce hypotension and mortality in mice may lead to toxic effects not seen at the lower doses that lead to sustained inflammatory responses in endotoxin-sensitive species such as humans [34]. In addition, interventions that protect rodents in models of endotoxin infusion may not be similarly protective in models of infection such as peritonitis [35].

The study has certain limitations. Cromolyn pretreatment was used to prevent confounding effects of mast cell degranulation and leakage during cremaster dissection, and so the experiments do not address the potential role of mast cells in mediating leakage during sepsis. In addition, responses were tested in only one vascular bed. Although cremaster videomicroscopy is a well studied microcirculatory model, and skeletal muscle comprises a good portion of total body mass in mice, some caution is warranted in extrapolating these results to other circulations. Because only one vessel was studied in each animal, the results also do not address regional heterogeneity in microvascular responses. This study was also not designed to assess microcirculatory flow and hematocrit, both of which may be important in sepsis; in making an assessment of these parameters, however, regional differences would be important, which would have necessitated a different study design. Finally, effects of anesthesia contributing to these observations cannot be ruled out. Inhalational isoflurane was titrated to the minimal doses required to maintain anesthesia, and septic mice required substantially lower doses than controls, but septic mice are likely to be more susceptible to the effects of anesthesia. The fact that vascular shear rates were comparable in septic and control animals suggests that anesthetic effects were reasonably similar. In addition, inhalational anesthetics have been shown to have anti-inflammatory effects, particularly after ischemia and reperfusion, but also during endotoxemia as well [36]. Because isoflurane was used twice, for performance of CLP and then again for cremasteric dissection, the possibility of isoflurane-induced preconditioning, which has been shown to influence microvascular leakage and iNOS induction [37], cannot be excluded. The anesthetic protocol was the same in both the septic and control groups, however, and a robust inflammatory response was observed with sepsis.

## Conclusion

Microvascular dysfunction causing intravascular leakage of fluid and protein contributes to hypotension and shock in sepsis. In a clinically relevant murine model of sepsis, we found discordance between adhesion of leukocytes and microvascular leakage, suggesting that these are regulated independently. These findings are pertinent to the mechanisms of vascular leakage in sepsis, and demonstrate that increased vascular permeability in sepsis is dependent on iNOS induction, but that leukocyte activation occurs with or without iNOS. Potential therapeutic implications include the possibility that selective iNOS inhibition may be a more promising approach than nonselective inhibition. The ability of NO to dilate blood vessels, block platelet and leukocyte adhesion to endothelial cells, and scavenge superoxide suggests that increased production of NO during sepsis acts to maintain microvascular blood flow and protect the endothelium from oxidative stress and damage. Attenuation of these protective effects by nonselective NOS inhibition could help explain the failure of this therapy when it is applied in patients with septic shock [38]. Translation of potential salutary effects of selective iNOS inhibition on vascular leakage to a measurable therapeutic benefit would require appropriately designed clinical trials.

## Key messages

• Leukocyte adhesion and microvascular leakage are both important contributors to hypotension and shock in sepsis. We showed that increased vascular permeability in sepsis is dependent on iNOS induction, but that leukocyte activation occurs with or without iNOS in a murine model. This suggests that leukocyte adhesion and microvascular leakage are regulated independently in sepsis

• Inhibition of iNOS ameliorated abnormal vascular permeability in septic mice. This suggests an important role for NO in mediating vascular leakage in sepsis.

• In addition to mediating vascular leakage, NO dilates blood vessels, blocks platelet and leukocyte adhesion to endothelial cells, and scavenges superoxide; these effects may maintain microvascular blood flow and protect the endothelium from oxidative stress and damage. Nonselective NO synthase inhibition has not proven to be an effective therapy in patients with septic shock, perhaps because of attenuation of these protective effects. Selective iNOS inhibition may be a more promising approach, but this hypothesis will need to be tested in appropriately designed clinical trials.

## Abbreviations

CLP = cecal ligation and puncture; iNOS = inducible nitric oxide synthase; NIH = National Institutes of Health; NO = nitric oxide; WBC = white blood cell.

## Competing interests

The authors declare that they have no competing interests.

## Authors' contributions

SMH conceived of the study; participated in its design, coordination and analysis; and helped to draft the manuscript. MG carried out the studies and participated in data analysis. JEP was involved in study design and helped to draft the manuscript. All authors read and approved the final manuscript.
